# An Examination of Two Policy Networks Involved in Advancing Smokefree Policy Initiatives

**DOI:** 10.3390/ijerph120911117

**Published:** 2015-09-08

**Authors:** Sarah Moreland-Russell, Bobbi J. Carothers

**Affiliations:** 1 George Warren Brown School of Social Work, Washington University in St. Louis, CB 1196, 1 Brookings Drive, St. Louis, MO 63130, USA; 2 Center for Public Health Systems Science, George Warren Brown School of Social Work, CB 1196, Washington University in St. Louis, 1 Brookings Drive, St. Louis, MO 63130, USA; E-Mail: bcarothers@wustl.edu

**Keywords:** public health policy, policy networks, smokefree, social network analyses

## Abstract

This study examines smokefree policy networks in two cities—Kansas City and St. Louis, Missouri—one that was successful in achieving widespread policy success, and one that was not. Descriptive social network analyses and visual network mapping were used to compare importance and contact relationships among actors involved in the smokefree policy initiatives. In Kansas City, where policy adoption was achieved, there was a higher level of connectivity among members, with network members being in contact with an average of more than five people, compared to just over two people for the St. Louis network. For both cities, despite being recognized as important, politicians were in contact with the fewest number of people. Results highlight the critical need to actively engage a variety of stakeholders when attempting city wide public health policy change. As evident by the success in smokefree policy adoption throughout Kansas City compared to St. Louis, closer linkages and continued communication among stakeholders including the media, coalitions, public health agencies, policymakers, and other partners are essential if we are to advance and broaden the impact of public health policy. Results indicate that the presence of champions, or those that play leadership roles in actively promoting policy by linking individuals and organizations, play an important role in advancing public health policy. Those working in public health should examine their level of engagement with the policy process and implement strategies for improving that engagement through relationship building and ongoing interactions with a variety of stakeholders, including policymakers.

## 1. Introduction

Over the past three decades, substantial progress has been made to control secondhand smoke exposure thanks to the steady increase in the adoption of comprehensive smokefree policies in localities across the U.S. [1]. Such policy development resulted from a collision of strong scientific evidence regarding the harms of smoking with the efforts of public health officials and grassroots advocates who stepped up as champions, set the policy agenda to “build the case for ‘nonsmokers’ rights”, and advanced the policy processes in localities and states across the nation [1].

Today, there are over 800 municipalities that have adopted some level of smokefree policies [2]. Most of these policies differ in the time and manner in which they were adopted. For example, the first smokefree policies adopted were not as comprehensive as smokefree policies initiated and adopted in later years. Also, while some smokefree policy battles were fought within state or local level legislatures, others were contended via ballot initiatives and decided by public vote [3]. Some smokefree policy initiatives arose from funded campaigns with national attention and strong opposition, others were quietly adopted with little deliberation, while other attempts suffered defeat. There are few other examples of great and lasting policy events like smokefree policy that offer an opportunity to study the complexity or possibility of the policy process in public health policy making.

The policy process is complex. It involves the coordination and collision of ideas, agendas, institutions, individuals, networks, and resources [4]. This complex process is also constantly evolving according to the physical, political, and social environment. In an effort to make sense of such chaos, Kingdon, in his Multiple Streams theory, offers a model that describes the interplay of these many pieces in the development of policy [4,5]. This multiple streams approach defines three distinct, but complementary, processes, or “streams”—problem, policy, and politics—that align to set the agenda and later converge, resulting in policy change. Though largely distinct in development and operation, these three streams can come together at critical times or “windows of opportunity”, where a problem is recognized, a solution is available, and the political climate makes the time right for change [4]. It is in this convergence of the three streams and resulting window of opportunity that allows for policy development and change.

Many scholars have recognized actors as integral in pushing for the coupling of streams, by defining problems and promoting solutions. According to the multiple streams framework, Kingdon subscribes to the notion that political institutions may make things possible, but people make things happen [4]. Similarly, the networks that form out of interpersonal and inter-organizational relationships among actors working on a similar policy have been found to be critical in facilitating the exchange of information regarding new ideas (or policy) and improving policy processes [6,7]. Credible policy networks and links have also been shown to serve as “platforms for action” [8,9], working to sway policy decisions.

Prior research has stressed the importance of studying policy networks and suggested further research focused on the role of policy networks in enhancing the policy process from agenda setting to policy adoption [7,10,11,12,13]. Whether in the form of individuals, organizations, or networks, people are defined as the main agents of change throughout the policy process and in communication across a system [4,10,11,14,15]. It is important to understand the relationships among these people as well as composition and structure of the resulting networks when examining the policy process.

Social network analysis has increasingly become a useful tool for examining public health systems [16,17,18,19,20,21,22]. The method is helpful for examining the relationships and connections between people and/or organizations. It can illustrate highly active partners and gaps in the network through the use of descriptive statistics and visualizations. Network analysis has been used frequently in tobacco control to study federal and state organizational relationships [16,18,19,23,24,25], yet has not been used broadly to discuss tobacco control policy networks in the U.S.

The purpose of this paper is to use social network analysis to examine two city-wide policy networks involved in similar public health policy initiatives focused in the area of tobacco control: (1) the Kansas City Smokefree Policy Initiative; and (2) The St. Louis Communities Putting Prevention to Work (CPPW) Smokefree Policy Initiative. The Kansas City smokefree policy campaign focused on the implementation of smokefree policies in cities throughout the Kansas City Metro Area. This initiative began with the inception of the CleanAir KC campaign in 2006 and was highly successful in achieving policy adoption—the campaign resulted in 22 out of 23 communities surrounding the Kansas City Metro Area adopting smokefree policies. The St. Louis CPPW initiative was funded as part of the American Recovery and Reinvestment Act of 2009. The St. Louis County Department of Health (StLDOH) was one of 50 communities awarded a CPPW grant. This initiative focused on implementing smokefree policies to reduce secondhand smoke exposure. Unfortunately, the St. Louis CPPW Smokefree Initiative was not successful in achieving comprehensive smokefree policy adoption. This paper examines and compares these separate networks, and presents similarities and differences between these policy networks. More specifically, we aim to understand the composition of and relationships among the actors in a policy network by examining importance and contact relationships.

## 2. Methods

The data collection methods employed for assessing network relationships for each city’s smokefree policy initiative are presented in Table 1. While data collection was slightly different for each assessment, we collected similar relational data for both. This section outlines the methods employed for each policy initiative. Human subjects’ exempt approval was obtained from the Institutional Review Board of Washington University in St. Louis for the study of both networks.

**Table 1:** Methods comparison for Kansas City and St. Louis Communities Putting Prevention to Work (CPPW) networks.

Measure	Kansas City SmokefreePolicy Campaign	St. Louis Smokefree Policy Initiative
*Data Collection*		
Mode	Semi-structured telephone interview	Online survey
Duration	60 min	15–45 min
Timeline	June 2010–August 2010	July 2011–September 2011
*Participants*		
Key Support	Advocacy, Government, Healthcare, Researchers	StLDOH Employees, Coalition Members, Leadership Team, Evaluation Team,
Partners	Community Members, Media, Other	Grantees
Politicians	City Council Members	County Council Members
*Network Measurements*		
Importance	Policy entrepreneurs are people within the community who play a leadership role in actively promoting a new policy, linking individuals and organizations and acting as positive advocates for the new policy. Based on this description, please identify up to 10 people who have been the most important to the success of policy adoption in your community.	We want to find out who you feel is most important to the completion of the CPPW activities. Please identify up to 15 people who you think are the *most important to the successful completion* of the above CPPW activities.
Contact	How often have you had direct contact (e.g., meetings, phone calls, faxes, letters, text/instant messages, or emails) with (insert name of policy entrepreneur or partner) during the time of SHS policy formation and adoption in your community? (Do not count listservs or mass emails). (0) Never, (1) Yearly, (2) Quarterly, (3) Monthly, (4) Weekly, or (5) Daily.	Please identify up to 20 people who you have had the *most contact with* (e.g., meetings, phone calls, faxes, letters, text/instant messages, or emails) regarding *CPPW activities in the last five or six months*. (Do not count listservs or mass emails. Do not include those receiving interventions.)

### 2.1. Kansas City Smokefree Policy Adoption Campaign

*Participants*. Social network analysis was incorporated to study three separate groups: (1) *politicians* (*i.e.*, city or county council members in the communities considering smokefree policy); (2) *key support* members defined as those identified as entrepreneurs or the most influential (non-policy maker) individuals in promoting policy and moving the smokefree policy process forward; and (3) *partners* defined as those involved at the grassroots or “boots on the ground” level in smokefree policy formation and adoption. An initial list of policy network members was generated by an examination of archival material, including CleanAir KC (local coalition) material, existing and archived news content, and archived city council hearing minutes in each of the 15 communities that adopted smokefree policies between April 2006 and April 2008 (*N* = 10). A one-stage snowball sample was used such that all additional individuals who were identified as politicians, key support members, or partners by the initial 10 participants were added to the network. Twenty-three people were selected during the second stage, 16 of whom agreed to participate. During both stages, a total of 53 people were identified. In order to account for the most relevant actors and to ensure a manageable sample size, only those persons nominated by more than one person were asked to participate in interviews. The interviews were conducted via phone by a trained research assistant and recorded to ensure accuracy in documenting responses.

*Measures*. The free-recall method was used to identify network members. Wording for *importance* and *contact* networks is presented in Table 1. For the importance network, participant responses were recorded as directed ties going from the respondent to the individuals they nominated. Since contact is an inherently reciprocal relationship (*i.e.*, if participant A says they are in contact with participant B on a monthly basis, participant B should say something similar about their contact with participant A), values for this network were symmetrized to form a non-directed network. Reciprocated ties were replaced with a single tie. In cases where pairs of participants responded with differing levels of contact for each other, the values were averaged. For pairs in which only one of the participants responded and data from the other actor was missing (either because they were mentioned only once and were not invited to participate (*n* = 27) or because they did not agree to participate (*n* = 8), reconstruction was used. For example, if participant A indicated monthly contact and participant B did not respond, participant A’s response was used and the link was coded as monthly contact. After symmetrizing, line values were dichotomized such that levels of communication at the quarterly level or above (responses 2 through 5) were retained and given a value of 1, and no communication or yearly communication (responses 0 and 1) were dropped. This cutoff was determined based on prior research incorporating similar relational constructs [23,24,25].

### 2.2. St. Louis CPPW Smokefree Policy Initiative

*Participants*. Three types of groups were included: (1) *politicians*, who were members of the St. Louis County Council; (2) *key support members,* who included St. Louis County Department of Health (StLDOH) employees, Tobacco-Free St. Louis Coalition board members, and members of the CPPW leadership team; and (3) *partners,* who included members of CPPW grantee organizations supported by the CPPW grant. An initial list of funded CPPW partners working specifically on the smokefree policy initiative included many of the key support members and partners (*n* = 71). A one-stage snowball sample was used such that all additional individuals who were named as important to the success of the completion of CPPW activities or were in contact regarding CPPW activities by the initial sample were also invited to participate (*n* = 73). Each individual who agreed to participate was sent an online Qualtrics survey. Additional individuals named by the second group were included in the analysis, but not invited to participate (*n* = 71). The total number of individuals included in the St. Louis network was 215.

*Measures*. The free-recall method was used to identify network members. Wording for *importance* and *contact* networks is presented in Table 1. For the importance network, participant responses were recorded as directed ties going from the respondent to the individuals they nominated. Since contact is inherently a bi-directional relationship, this network was symmetrized such that if one or both members of a pair indicated a tie, it was counted as such. Unlike Kansas City, the contact network in St. Louis was binary (present or not) instead of valued, so consideration of value differences between pairs was unnecessary.

### 2.3. Analysis for Both Networks

Descriptive social network analyses were conducted for the importance and contact networks. For the importance networks, in-degree (number of incoming nominations) was calculated for each person. Average in-degree was then calculated for the overall network, as well as for key support, partners, and politicians. For the contact networks, degree (number of connections) was calculated for each person. Average degree was then calculated for the overall network, as well as for key support, partners, and politicians. Betweenness centrality (a measurement of how often a person connects to others who are otherwise not connected) was also calculated for each person in the contact networks [26]. It was averaged over key support, partners, and politicians as an additional measure of how central the three different kinds of people were relative to each other. Other common network measures, (*i.e.*, density, degree centralization, and betweenness centralization) were not calculated because they are heavily influenced by network size [27], and the St. Louis network was more than four times the size of the Kansas City network. The analyses and network visualizations were created with Pajek [28]. Node sizes in the visualizations was determined by in-degree for the importance networks and by degree in the contact networks.

## 3. Results

The Kansas City Network included 53 people—13 as key support, 28 as partners, and 12 as politicians. Out of these 53 members, 33 were asked to participate in an interview and 26 agreed with a response rate of 78%. The St. Louis Network included 215 people—113 as key support, 94 as partners, and 8 as politicians. For the St. Louis network, out of the 215 people, 145 were invited to participate and 74 responded, for a response rate of only 51%, below the 70% desired for social network analysis [29].

Table 2 shows the average number of links for each of the entire networks, as well as for key support, partner, and politician members. Overall, the Kansas City network demonstrated a higher level of connectivity for contact, with members being in contact with an average of more than five people, compared to just over two people for the St. Louis network. For Kansas City, key support members received the greatest number of nominations for being important on average (3.23), followed by politicians (2.83) and partners (2.07). For St. Louis, politicians received the greatest number of importance nominations on average (9.13), followed by partners (1.36) and key support members (1.35). For both cities, politicians were in contact with the fewest number of people.

There were also noted differences among the two networks in the kinds of individuals who were most important connecting others (*i.e.*, betweenness centrality) within the network. For Kansas City, betweenness centrality in the contact network was highest for key support members, followed by politicians and partners. For St. Louis, betweenness centrality was highest for the partner and key support members. None of the St. Louis politicians had a betweenness centrality score above zero.

**Figure 1:**
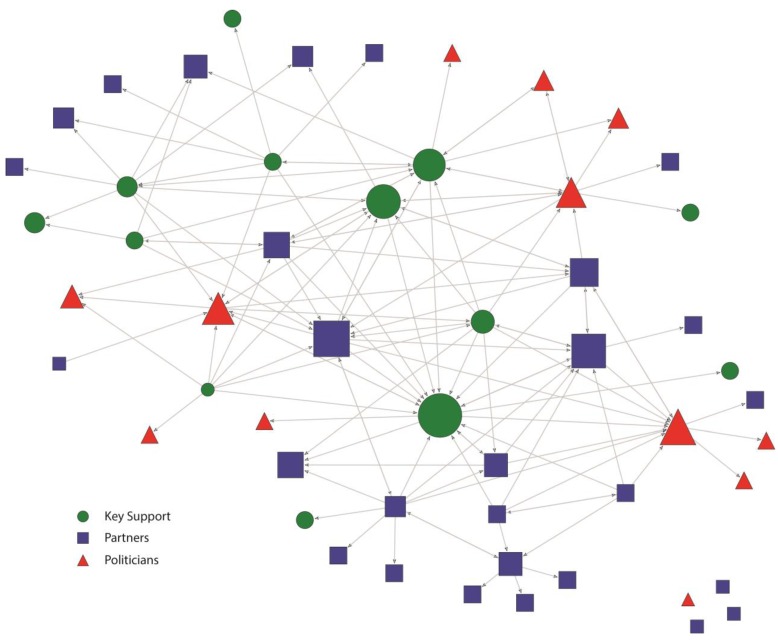
Kansas City Smokefree Policy Importance network. Nodes are sized according to the number of importance nominations. Links show direction of importance nominations.

**Figure 2:**
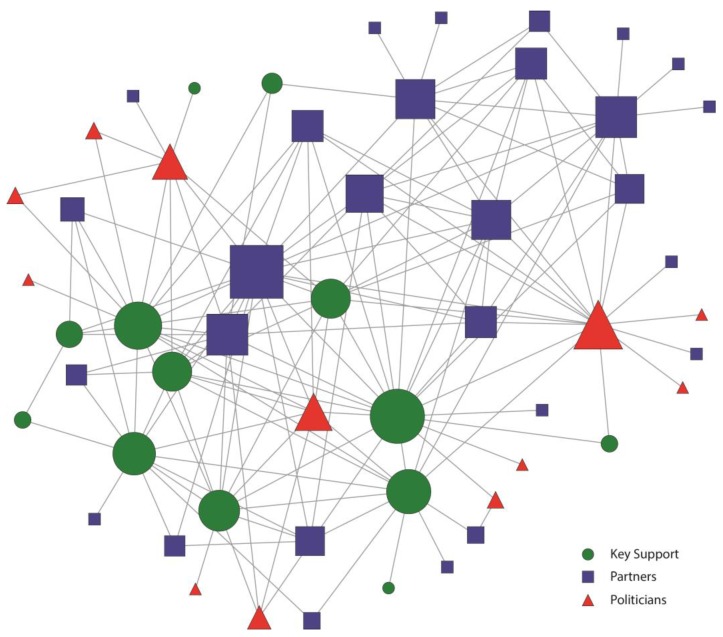
Kansas City Smokefree Policy Contact network. Nodes were sized by the number of individuals they had contact with.

**Figure 3:**
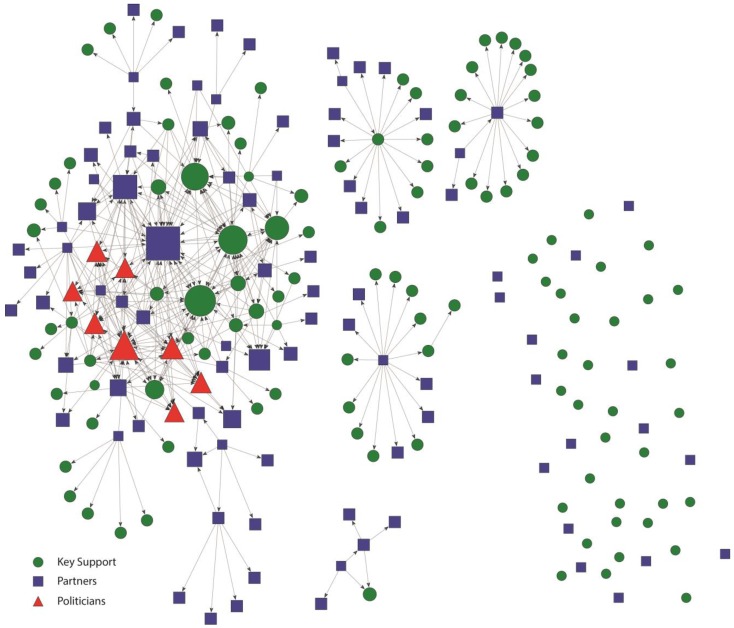
St. Louis Smokefree Policy Importance network. Nodes are sized according to the number of importance nominations. Links show direction of importance nominations.

**Figure 4:**
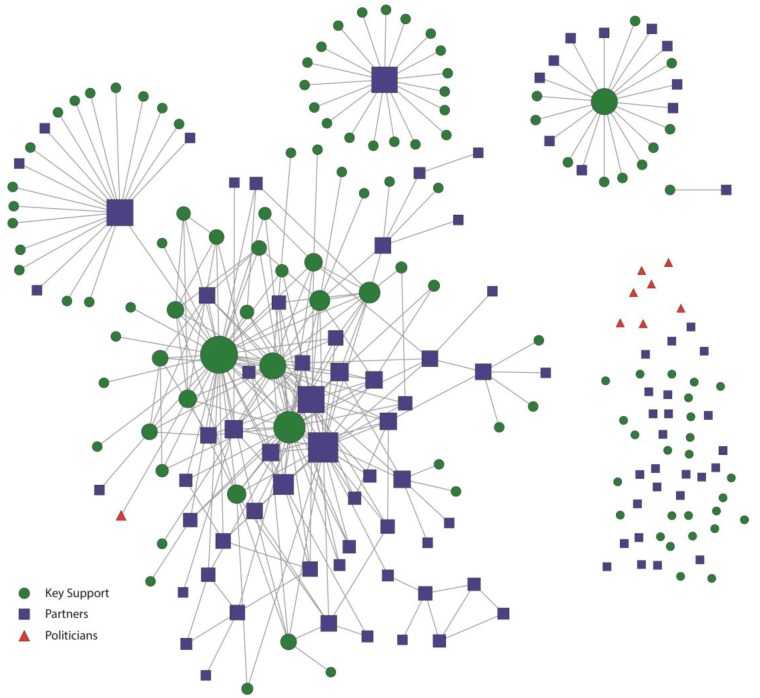
St. Louis Smokefree Policy Contact network. Nodes were sized according to the number of individuals they had contact with.

These patterns are also evident in the network maps presented in Figure 1, Figure 2, Figure 3 and Figure 4. Figure 1 and Figure 2 represent the structure of importance and communication as assigned by the members in the Kansas City network, respectively. Figure 3 and Figure 4 represent the structure of importance and communication among the members in the St. Louis network, respectively. The shapes in each figure represent whether the member was identified as a politician, key support member, or partner. The size of the nodes represents the member’s in-degree or degree (*i.e.*, those with more connections had larger nodes).

**Table 2:** Size, average degree, and average betweenness centrality measures for entire networks, key support, partner, and politician members.

Measure	Network Size	Average Degree Overall	Average Degree			Average Betweenness Centrality		
			Key Support	Partner	Politician	Key Support	Partner	Politician
*Kansas City*								
Importance ^1^	53	2.53	3.23	2.07	2.83			
Contact	53	5.66	8.62	4.89	4.25	0.056	0.019	0.024
*St. Louis*								
Importance ^1^	215	1.65	1.35	1.36	9.13			
Contact	215	2.68	2.50	3.12	0.13	0.004	0.004	0.000

^1^ In-degree reported for Importance networks.

In both cities, politicians were seen as important (Figure 1 and Figure 3) as indicated by their relatively large size. Importance in the Kansas City network was shared across key support, politician, and partner members as demonstrated by their relatively even distribution through the network (Figure 1), with only four members not nominated (*i.e.*, 4 isolates). This reflects shared buy-in and recognition of many individuals as serving an important role in the Kansas City smokefree policy process. Importance in St. Louis was more heavily focused on politicians, as demonstrated by their more central location as compared to Kansas City (Figure 3), and receiving a greater share of the nominations (Table 2). The disconnected components in the St. Louis importance networks were mostly nominations within isolated partner grantee organizations.

Contact was also very different among members in each of the networks (Figure 2 and Figure 4). In the Kansas City smokefree contact network (Figure 2), key support members were key in establishing many connections and serving as the main source of contact for some of the other members. These key support members represented a variety of government public health offices, healthcare organizations (*i.e.*, physicians), the smokefree coalition (CleanAir KC), and the media. They were all well integrated into the network and were central to communication, making them hubs of information, important in linking actors that would not otherwise be connected. In addition, there were no isolates in the Kansas City contact network showing an actively engaged and cohesive network. Partners were also important and active members of the Kansas City contact network. All of the politicians maintained contact with at least one other network member, even if most of them were located on the periphery of the network.

The St. Louis smokefree policy contact network (Figure 4) was much less connected in comparison to the Kansas City network. First, there were several isolates and many separate small components comprised of members who were not well connected to the main component. Disconnected components in the St. Louis contact network mostly included relationships within grantee partner organizations. Partners and key support members with the highest centrality measures represented the government public health office and the smokefree coalition. However, even those with highest centrality were limited in their level of engagement with many other members in the network. Unlike the Kansas City communication network, media partners were not well integrated into the St. Louis communication network. The politicians demonstrated almost no contact in the St. Louis smokefree policy network. Only one politician was connected, demonstrating that the other politicians did not communicate with any network members. This one politician was only in contact with one other network member, a member of the smokefree coalition.

## 4. Discussion

This study highlights how the policy process can be shaped by networks by examining policy network in two cities—one which was successful in achieving widespread policy success (Kansas City) and one that was not (St. Louis). Overall, the Kansas City network was more connected than the St. Louis network. For both cities, the relative level of importance of politicians was greater than their contact. In addition, members in the Kansas City’s smokefree policy network were well connected and actively communicated with all other members while the St. Louis smokefree policy network was not well connected.

This study is the first to document and track two separate policy networks working on similar public health policy initiatives in two distinct cities within the same state. Examination of these two networks and the outcomes of the policy processes provide insight into the actors and relationships important in achieving policy success. First and most importantly, results from this study highlight the critical need to actively engage a variety of stakeholders when attempting city wide public health policy change. While each of the city’s networks included a wide variety of stakeholders, they differed in the engagement of the stakeholders. For instance, the Kansas City contact network was completely linked and had no isolates, indicating that all members were actively engaged and sharing information throughout the initiative. The St. Louis network on the other hand contained several isolates—even among partners and key support members—indicating that there was not active engagement of all stakeholders. Studies have shown that engagement of all members through active communication and information sharing can bypass formal barriers to consensus and establish trust among network actors [10,11,13]. Kansas City’s active communication and involvement of all members may have been a contributing factor to its policy success.

In addition, it is important to have several champions that play leadership roles in actively promoting a new policy by linking individuals and organizations and by acting as positive advocates for the new policy [11,14]. Research on community champions shows that they are unique in having extensive social contacts and negotiating skills and that they may be found at various levels of status in community [6,11,14,30]. In Kansas City, we found that there were several members who exemplified these “champion” characteristics and who were recognized as important in moving smokefree policy forward. Most importantly, these same members were also central to the communication network with many contacts and ability to connect many other stakeholders. In St. Louis, it is harder to identify similar champions as those recognized as important were often not those who were central in the communication network.

Finally, engaging policymakers also seemed evident in advancing policy. In Kansas City, politicians in all but one of the local communities were engaged in the network, even if only through the contact of key support members. St. Louis on the other hand recognized the importance of politicians in achieving policy change, however did not regularly communicate with these political champions. This disengagement of policy makers could have contributed to the lack of policy success in St. Louis.

Many of our findings are consistent with previous tobacco literature outlining the critical role of local level partnership and collaboration networks (often in the form of tobacco control coalitions) in achieving tobacco control policy success. Through the review of tobacco industry documents and study of tobacco control policy efforts at state and local levels, tobacco control scientists have established the influence of the tobacco lobby in preventing statewide smokefree policy legislation, including providing campaign contributions to legislators and funding opposition to state level tobacco control policy [31,32]. This overt opposition led local level tobacco control activists to form networks and build the smokefree policy movement from the grassroots. For over three decades, tobacco control coalitions at the local level have mobilized their communities to participate in tobacco control efforts, combat the tobacco industry, and change the culture around tobacco. The most successful coalitions were formed at the local level, included interpersonal networks comprised of diverse stakeholders, and engaged important policy or decision makers [14,32,33].

Some limitations of this study should be acknowledged. First, this study compares two city wide smokefree policy efforts initiated at different times for different reasons. The Kansas City network was born out of a grassroots effort based on broad community support and desire for citywide smokefree policy adoption. In contrast, the St. Louis network emanated because of funding with outlined objectives directed toward achieving a comprehensive smokefree policy effort. While we feel there is much to be learned in the types of members included in the network and the connections among them, it is important to acknowledge these differences.

Another important concern in a social network study is which actors to include [26]. For Kansas City, network membership was determined by the identification of other actors in the network. This is a realist approach [34] in which actor membership and boundaries are perceived by the actors themselves. While this approach is considered to be useful in determining network boundaries, it relies heavily on participant recall, possibly limiting network membership. For St. Louis, we relied on the primary recipients of CPPW funding to define partners and verified the partnership lists with each grantee. To overcome this potential limitation, we employed a one-stage snowball sample methodology to identify additional individuals who were named as important to the success of the completion of CPPW activities. However, the participation rate from the St. Louis group was only 51%, below the 70% desired for social network analysis [29]. Given the low response rate and its particular implications for network analysis [35], the results for the St. Louis network should be interpreted with some caution. It is important to recognize other factors that might have contributed to the lower response rate. As a national initiative, CPPW was met with resistance [36], and this national level attention could be related to the Council’s lack of engagement with the CPPW network, as well as the failure to pass a comprehensive smokefree policy in St. Louis.

We used previously published methods [16,22] to account for non-responders and symmetrized data to an average level of contact for the Kansas City network and any contact for the St. Louis network. This may have increased the number of network ties, but would do so for both networks. We feel it is more likely that one partner would remember a relationship that the other forgot, rather than mistakenly recall a relationship that did not exist. One final criticism is the use of different network measures in the comparison of the Kansas City and St. Louis networks. Therefore, direct comparison of the network statistics in Table 2 should be done with caution. However, the overall conclusion remains the same: a strong, well connected network was related to successful policy change, and speaks to the possible convergent validity of the measures used.

In summary, strong interconnected policy networks like that seen in the Kansas City smokefree policy network are important in public health policy development and adoption. Those working in public health should examine their level of engagement with the policy process and implement strategies for improving that engagement through relationship building and ongoing interactions with a variety of stakeholders including policymakers.
